# Flavonoids and alkaloids from the rhizomes of *Zephyranthes ajax* Hort. and their cytotoxicity

**DOI:** 10.1038/s41598-020-78785-2

**Published:** 2020-12-17

**Authors:** Khan Viet Nguyen, Duc Viet Ho, Nhan Trong Le, Kiem Van Phan, Jyrki Heinämäki, Ain Raal, Hoai Thi Nguyen

**Affiliations:** 1 Faculty of Pharmacy, Hue University of Medicine and Pharmacy, Hue University, 06 Ngo Quyen, Hue, Vietnam; 2 Institute of Marine Biochemistry, The Vietnam Academy of Science and Technology, Hanoi, Vietnam; 3 Institute of Pharmacy, Faculty of Medicine, University of Tartu, 1 Nooruse str., 50411 Tartu, Estonia

**Keywords:** Cancer, Chemical biology, Plant sciences, Medical research, Oncology

## Abstract

A new flavanol derivative, (2*R*,3*R*)-3-acetoxy-7-hydroxy-3′,4′-methylenedioxyflavan (**1**), was co-isolated from the rhizomes of *Zephyranthes ajax* Hort. with the following seven known compounds: 7-hydroxyflavan (**2**), 7,4′-dihydroxyflavan (**3**), 7,4′-dihydroxy-8-methylflavan (**4**), 7,3′-dihydroxy-4′-methoxyflavan (**5**), 5,4′-dihydroxy-7-methoxy-6-methylflavan (**6**), 7-hydroxy-3′,4′-methylenedioxyflavanone (**7**) and haemanthamine (**8**). Their structures were elucidated by combining 1D-/2D-NMR, CD, UV and HRESIMS data, and comparisons with reported data in literature were made. Among these known compounds, **2**, **3**, **4**, **6** and **7** were isolated from the genus *Zephyranthes* for the first time. In addition, the cytotoxicity assay indicated that compound **8** has potent cytotoxic activity against human hepatocellular carcinoma (the HepG2 cell line), human lung carcinoma (the SK-LU-1 cell line), human carcinoma in the mouth (the KB cell line), human colon carcinoma (the SW480 cell line) and human stomach gastric adenocarcinoma (the AGS cell line), with IC_50_ values ranging from 4.4 to 11.3 µM. This is the first study reporting the cytotoxicity of compound **8** against the SK-LU-1 cancer cell lines.

## Introduction

The plants of the family Amaryllidaceae consist of ca. 85 genera and 1100 species widely distributed in the temperate and tropical regions of the globe^1^. Many species of this family have been used as remedies for inflammation, circulatory and neurological diseases^2^. Amaryllidaceae species contain numerous alkaloids with proven significant medical value. According to literature, there are over 500 Amaryllidaceae alkaloids possessing acetylcholinesterase inhibitory, cytotoxic, antitumor, analgesic, antifungal, antimalarial, antiviral and antibacterial activities^2,3^.

*Zephyranthes ajax* Hort*.* is an amaryllidaceous bulbous perennial. Bulbs are around 1 cm in diameter. Leaves are green, smooth and have linear blades which are around 30 cm long and 0.4–0.5 cm wide. Flowers are yellow and they have six petals (each 2 cm long) and equal-length stamens (around half of the petal length). *Z. ajax* is mainly used as an ornamental plant in Vietnam^4^. To date, however, the knowledge on the chemical constituents of *Z. ajax* is still limited. Phytochemical studies have revealed that this genus contains many alkaloids with cytotoxic, acetylcholinesterase inhibition, antiviral and antibacterial activity^5–8^. In addition, the genus contains flavonoids, flavans, gibberllins, phospholipids, sterols, lectins and terpenoids^7^. In this paper, we describe the isolation, structure elucidation and cytotoxic activities of a new flavanol derivative (**1**) and seven known compounds (**2**–**8**) found from *Z. ajax* collected in Vietnam.

## Results and discussion

**Figure 1:**
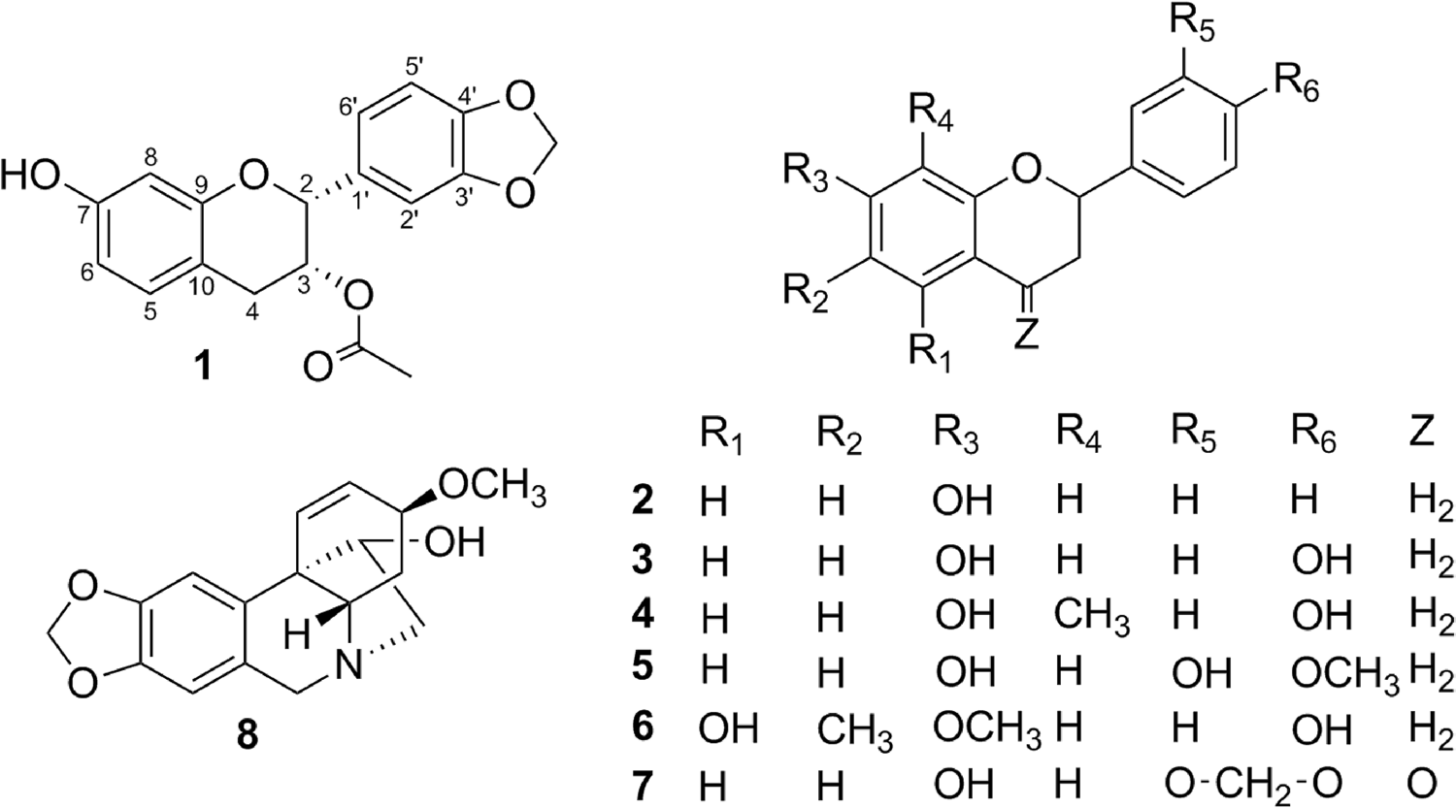
Structures of **1**–**8** isolated from *Zephyranthes ajax* Hort.

The structures of known compounds were established by means of spectroscopic (^1^H-, ^13^C-NMR and MS) and these results were in good agreement with the previous studies, including 7-hydroxyflavan (**2**)^9^, 7,4′-dihydroxyflavan (**3**)^10^, 7,4′-dihydroxy-8-methylflavan (**4**)^11^, 7,3′-dihydroxy-4′-methoxyflavan (**5**)^12^, 5,4′-dihydroxy-7-methoxy-6-methylflavan (**6**)^13^, 7-hydroxy-3′,4′-methylenedioxyflavanone (**7**)^14^ and haemanthamine (**8**)^15,16^ (Fig. 1). To our best knowledge, this is the first study reporting the presence and isolation of compounds **2**, **3**, **4**, **6** and **7** in the genus *Zephyranthes*.

**Figure 2:**
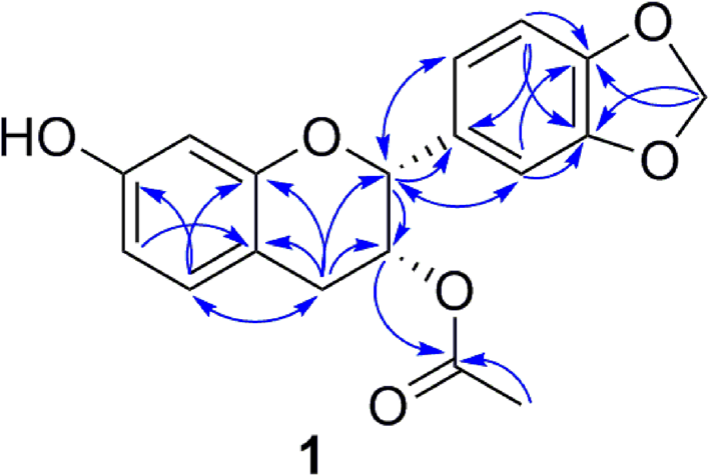
Key HMBC (^1^H → ^13^C, arrows) correlations of **1**.

Compound **1** was obtained as a colourless powder. The high-resolution electron spray ionization mass spectrometry (HRESIMS) of **1** showed a quasi-molecular ion peak at *m/z* 329.1021 [M+H]^+^. Based on the HRESIMS and NMR data, its molecular formula is evidently C_18_H_16_O_6_, requiring eleven degrees of unsaturation. The ^1^H NMR spectrum of **1** exhibited the characteristic signals corresponding to two sets of ABX patterns at δ_H_ 6.90 (d, *J* = 8.5 Hz, H-5), 6.40 (dd, *J* = 8.5, 2.5 Hz, H-6), 6.37 (d, *J* = 2.5 Hz, H-8) and δ_H_ 6.98 (d, *J* = 1.0 Hz, H-2′), 6.82 (d, *J* = 8.0 Hz, H-5′), 6.95 (dd, *J* = 8.0, 1.0 Hz, H-6′), belonging to two 1,3,4-trisubstituted benzene rings. In addition, the signals of a dioxygenated methylene group at δ_H_ 5.97 (s), two oxygenated methine groups at δ_H_ 5.11 (s, H-2), 5.37 (m, H-3) and an acetoxy group at δ_H_ 1.89 (s) were observed. The acetoxy methyl protons resonate at higher field due to the anisotropic effect of two aromatic rings. This observation is in accordance with previous studies on 3-acetoxyflavanol derivatives^17,18^. The ^13^C NMR, DEPT and HSQC spectra of **1** revealed 18 signals including a carbonyl carbon (δ_C_ 171.9), twelve aromatic carbons (δ_C_ 158.0, 156.2, 149.0, 148.7, 133.7, 131.3, 121.0, 110.8, 110.0, 108.8, 108.1, 104.0), a dioxygenated methylene carbon (δ_C_ 102.4), two oxygenated methine carbons (δ_C_ 78.4, 70.2), a methylene carbon (δ_C_ 31.1) and a methyl carbon (δ_C_ 20.8) (Table 1). The present results suggest that compound **1** is a flavanol derivative. The planar structure of **1** was established by detailed HMBC analysis (Fig. 2). Notably, the HMBC cross-peak from H-3 (δ_H_ 5.37) to carbonyl carbon (δ_C_ 171.9) led to the introduction of acetoxy group at C-3. This was supported by the strong downfield shifts of C-3 (δ_C_ 70.2), H-3 (δ_H_ 5.37) compared to those of (2*R*,3*R*)-3,7-dihydroxy-3,4-methylenedioxyflavan (δ_C_ 67.1, δ_H_ 4.21)^19^.

**Table 1.** ^1^H (500 MHz) and ^13^C (125 MHz) NMR data of **1** in methanol-*d*_*4*_ [δ (ppm), *J* (Hz)].

Position	*δ*_C_	*δ*_H_
2	78.4	5.11 s
3	70.2	5.37 m
4	31.1	2.81 dd (17.0, 2.0) (H-4a) 3.25 dd (17.0, 4.0) (H-4b)
5	131.3	6.90 d (8.5)
6	110.0	6.40 dd (8.5, 2.5)
7	158.0	–
8	104.0	6.37 d (2.5)
9	156.2	–
10	110.8	–
1′	133.7	–
2′	108.1	6.98 d (1.0)
3′	149.0	–
4′	148.7	–
5′	108.8	6.82 d (8.0)
6′	121.0	6.95 dd (8.0, 1.0)
–OAc	171.9	–
	20.8	1.89 s
–OCH_2_O–	102.4	5.97 s

Assignments were done by HSQC, HMBC experiments.

The CD spectrum of **1** indicated a negative Cotton effect at λ 288 nm (^1^L_b_ band) which was associated with *P*-helicity of heterocyclic C ring. Therefore, the absolute configuration of C-2 was determined as *R* form^20^. Furthermore, the small vicinal coupling constant values (*J*_H-2/H-3_ = 0 Hz, *J*_H-3/H-4a_ = 2.0 Hz, *J*_H-3/H-4b_ = 4.0 Hz) were indicative of the *cis* relationship between H-2 and H-3^21^. Based on the above evidences, compound **1** was elucidated to be (2*R*,3*R*)-3-acetoxy-7-hydroxy-3′,4′-methylenedioxyflavan.

The cytotoxicity of all isolates was initially evaluated against human hepatocellular carcinoma (the HepG2 cell line) and human lung carcinoma (the SK-LU-1 cell line). As shown in Table 2, compounds **1**–**7** did not show any cytotoxicity against the two tested cancer cells. Compound **8** exhibited potent activity against the HepG2 and SK-LU-1 cell lines, with IC_50_ values of 9.7 and 5.4 µM, respectively. Particularly, the cytotoxic activity against the SK-LU-1 cancer cell line of **8** has not been reported previously. Based on the initial results, compound **8** was selected for the further investigation against human carcinoma in the mouth (the KB cell line), human colon carcinoma (the SW480 cell line) and human stomach gastric adenocarcinoma (the AGS cell line). As expected, compound **8** showed a strong inhibitory effect towards the KB, SW480 and AGS cell lines, with IC_50_ values of 11.3, 4.4 and 6.5 µM, respectively. These findings are in agreement with those reported in literature^22,23^. According to Havelek et al., compound **8** acts as a potential anticancer agent owing to the decrease in cell viability and induction of apoptosis accelerating the caspases activation. With treatment of **8** in Jurkat cells, caspase-9 and caspase-3/7 activation are more intense and caspase-8 activation was less intense, in addition to the arrest of the cell cycle and the decrease in mitochondrial membrane potential which plays a crucial step in the apoptotic process. Furthermore, the agent is able to stimulate DNA damage checkpoint kinase Chk1 and the p16^INK4a^ cyclin-dependent kinase inhibitor^24^. Pellegrino et al. reported that compound **8** is capable to form complex with the *Saccharomyces cerevisiae* 80S ribosome through binding at the A-site cleft of the peptidyl tranferase centre on the large ribosomal subunit, thus generating specific molecular interactions with the 25S rRNA^25^. The authors found that that ribosomes are bound by one molecule of compound **8** at a time, thus enabling highly specific drug targeting. Moreover, compound **8** was found to form a sandwich-like structure between the two 25S rRNA redidues (U2875 and C2821). The accommodation of compound **8** in the present structure is also stabilized by the formation of two additional hydrogen bonds associated with the C11-hydroxyl group^25^. These result in the halting of the elongation phase of eukaryotic translation. Consequently, haemanthamine treatment specifically restrains ribosome biogenesis, activates nucleolar stress response and stabilises p53 in cancer cells, which are responsible for the preferential killing of cancer cells^25^. Compound **8** has been proven to have a cytotoxic activity, but it is very poorly soluble in water. Therefore, we recently developed novel haemanthamine (compound **8**)-loaded amphiphilic nanofibers to overcome these formulation challenges^26^. Noticeably, secondary metabolites from natural products have been the most successful leads in the discovery and development of novel drugs^27,28^. However, these plant-origin compounds possess a number of disadvantages associated with their large molecule size, poor water solubility, poor oral bioavailability, limitations in target-specific drug delivery, and in-vivo instability^29,30^. Hence, the development of novel drug delivery systems for natural compounds could resolve these critical issues, thus leading to novel and versatile applications of natural products in medicine. In our recent study, we succeeded to yield (through extraction and crystallization) haemanthamine (compound **8**) in the amounts enough to fabricate the active-loaded amphiphilic nanofibers^26^. The results suggested that the formulation of such amphiphilic nanofibers for haemanthamine could provide a promising drug delivery system for the present large-molecule and poorly water-soluble active agent.

**Table 2:** Cytotoxicity of the compounds **1**–**8** isolated from *Z. ajax* Hort. and ellipticine (as a positive control).

Compound	IC_50_ (µM) ± SD				
	HepG2	SK-LU-1	KB	SW480	AGS
**1**	> 100	> 100	N.T.	N.T.	N.T.
**2**	> 100	> 100	N.T.	N.T.	N.T.
**3**	> 100	> 100	N.T.	N.T.	N.T.
**4**	> 100	> 100	N.T.	N.T.	N.T.
**5**	> 100	> 100	N.T.	N.T.	N.T.
**6**	> 100	> 100	N.T.	N.T.	N.T.
**7**	> 100	> 100	N.T.	N.T.	N.T.
**8**	9.7 ± 0.7	5.4 ± 0.2	11.3 ± 0.9	4.4 ± 0.1	6.5 ± 0.7
Ellipticine	1.5 ± 0.1	1.6 ± 0.1	1.5 ± 0.1	1.5 ± 0.1	0.7 ± 0.1

*N.T.* the compounds were not tested.

## Materials and methods

### General experimental procedures

A JASCO P-2000 polarimeter (Hachioji, Tokyo, Japan) was used to record optical rotation. UV spectra were measured using a Shimadzu UV-1800 spectrophotometer (Shimadzu, Kyoto, Japan). Circular dichroism spectra were studied with a Chirascan CD spectrometer (Applied Photophysics Ltd., Surrey, United Kingdom). A Bruker Avance 500 spectrometer (Bruker, MA, USA) was exploited for investigating NMR spectra and TMS as an internal reference. HRESIMS data were performed using an Agilent 6530 Accurate-Mass spectrometer (Agilent, CA, USA). The column chromatography studies were conducted with YMC RP-18 (Fuji Silysia Chemical Ltd, Kasugai, Aichi, Japan), Cosmosil 75C18-OPN (Nacalai Tesque Inc., Kyoto, Japan), silica gel (40–50 µm, Kanto Chemical Co., Tokyo, Japan), Diaion HP-20 (Mitsubishi Chem. Co., Tokyo, Japan) and Sephadex LH-20 (Dowex 50WX2-100, Sigma–Aldrich, USA). Pre-coated silica gel 60F_254_ and RP-18 F_254_ plates (0.25 or 0.50 mm thickness, Merck, Germany) were exploited to investigate analytical TLC. Preparative HPLC with a DAD detector applied was Agilent 1260 Infinity II system (Agilent, CA, USA) and using a Zorbax SB–C_18_ column (5 µm particle size, 9.4 × 250 mm).

### Plant material

The whole plants of *Z. ajax* were collected in Hue city, Vietnam (geographical coordinates: 16°27′43.8″N; 107°33′55.4″E) in May 2017. The plant material was authenticated by Dr Chinh Tien Vu (Vietnam National Museum of Nature, VAST, Vietnam). A voucher specimen has been stored at the Faculty of Pharmacy, Hue University of Medicine and Pharmacy, Hue University, Vietnam.

### Extraction and isolation

The powdered bulbs (cut from the ground parts) of *Z. ajax* (5.5 kg) were extracted three time with MeOH (10.0 L each, at room temperature) to obtain 467 g extract. After suspension of the extract using water (2.0 L), partition was conducted with dichloromethane, ethyl acetate, *n*-butanol (3 times, 2.0 L each). The solvents were removed *in vacuo* to yield the dichloromethane (D, 120.3 g), ethyl acetate (E, 126.8 g), *n*-butanol (43.5 g) and water (140.3 g)-soluble portions.

The D extract was loaded onto a silica gel column, eluted with a *n*-hexane–acetone gradient solvent system (100:0, 95:5, 90:10, 50:10, 10:10, 0:100 v/v, each 1.5 L to get 6 fractions D1–D6). Fraction D3 (20.5 g) was subjected to a silica gel column using *n*-hexane–ethyl acetate (8:1, v/v) as an eluent to give 8 sub-fractions (D3.1–D3.8). Fraction D3.2 (2.3 g) was chromatographed with a RP-18 column chromatography eluting with MeOH–water (4:1, v/v) to give 10 fractions (D3.2.1–D3.2.10). Fraction D3.2.5 (212 mg) was then applied to a Sephadex LH-20 column by eluting with MeOH to give 3 fractions (D3.2.5.1–D3.2.5.3). Fraction D3.2.5.2 (78 mg) was separated by preparative reversed-phase HPLC using acetonitrile–water (75:25, flow rate 2.0 mL/min) as an eluent to obtain **1** (4.2 mg) and **2** (3.7 mg). Fraction D3.4 gave a precipitate. After filtering and drying, the precipitate was dissolved in CHCl_3_–MeOH (1:1, v/v), and then recrystallised to yield **8** (210 mg) as a white precipitate.

The E extract was chromatographed on a silica gel column using *n*-hexane–acetone gradient solvent system (100:0, 75:10, 50:10, 25:10, 10:10, 0:100 v/v, each 1.5 L to get 6 fractions E1–E6) as the eluent. Fraction E4 (18.5 g) was further fractionated on a silica gel column by eluting with *n*-hexane–ethyl acetate–acetone (10:1:1, v/v) to yield 7 fractions (E4.1–E4.7). Fraction E4.2 (2.1 g) was subsequently purified with a RP-18 column by eluting with MeOH–water (3:1, v/v) to get 6 fractions (E4.2.1–E4.2.6). Fraction E4.2.4 (281 mg) was further separated by a Sephadex LH-20 column by eluting with MeOH–water (4:1, v/v) to give 4 sub-fractions (E4.2.4.1–E4.2.4.4). Fraction E4.2.4.2 (94 mg) was purified by preparative reversed-phase HPLC using acetonitrile–TFA in water 0.05% (65:35, flow rate 2.0 mL/min) as an eluent to obtain **3** (3.0 mg), **4** (3.8 mg). Fraction E4.4 (2.1 g) was further applied to a silica gel column and eluted with *n*-hexane–CH_2_Cl_2_–MeOH (5:1:1, v/v) to get 5 fractions (E4.4.1–E4.4.5). Fraction E4.4.3 (451 mg) was further fractionated with a Sephadex LH-20 column by eluting with MeOH to yield 4 fractions (E4.4.3.1–E4.4.3.4). Fraction E4.4.3.3 (143 mg) was further purified by preparative reversed-phase HPLC and eluted with MeOH–water (73:27, flow rate 2.0 mL/min) to produce **5** (4.1 mg), **6** (2.9 mg) and **7** (4.4 mg).

(2*R*,3*R*)-3-Acetoxy-7-hydroxy-3′,4′-methylenedioxyflavan (**1**): Colourless powder; ${[}\alpha {]}_{D}^{25}$ − 18.0 (*c* 0.1, MeOH); UV (MeOH) *λ*_max_ (nm): 203, 284, 288; CD (MeOH) *λ*_max_ (nm) (Δε, mdeg.): 206 (− 4.07), 224 (− 0.52), 231 (− 0.73), 252 (+ 0.05), 288 (− 0.45), 341 (− 0.15); ^1^H and ^13^C NMR: see Table 1; HRESIMS found: *m/z* 329.1021 [M+H]^+^ (calcd. for C_18_H_17_O_6_, 329.1025).

### Cytotoxicity assay

The effects of compounds **1**–**8** on the growth of human cancer cells, including HepG2 and SK-LU-1, were tested using a sulforhodamine B assay^31,32^. Two human cancer cell lines were grown in a Dulbecco’s modified eagle medium (DMEM) consisting of 2.0 mM l-glutamine, 10.0 mM HEPES and 1.0 mM sodium pyruvate. The DMEM was supplemented with 10% foetal bovine serum, FBS (GIBCO). The cells were sub-cultured every 3–5 days and kept in a humidified atmosphere containing 5% CO_2_ at 37 °C. Subsequently, the cells were detached using 0.05% Trypsin–EDTA. A sulforhodamine B (SRB) method based on the determination of cellular protein content was exploited to assess the proportion of viable cells in a cell population. In 96-well microplates, viable cells were cultured overnight (4 × 10^4^ cells/well) in 180 μL of growth medium. The cells were then treated with tested samples at various concentrations of 100, 20, 4 and 0.8 μg/mL and maintained under the same conditions for 3 days. After removing the medium, cold 20% (w/v) trichloroacetic acid was used to fix the remaining cell monolayers for 1 h at 4 °C. A 1X SRB staining solution was applied to stain the fixed cells for 30 min. Subsequently, 1% (v/v) acetic acid was applied three times to remove the unbound dye. ELISA Plate Reader (Bio-Rad) was used for the absorbance measurement (at 515 nm) of the protein-bound dye dissolved in a 10-mM Tris base solution. DMSO 10% and ellipticine were used as a negative control and positive control, respectively. The half maximal inhibitory concentration (IC_50_) was determined using TableCurve Version 4.0 (Systat Software, Inc., USA)^33,34^. All experiments were performed in triplicate.

### Supporting information

HRESIMS, UV, CD and NMR spectra (^1^H and ^13^C NMR, DEPT, HSQC, HMBC) for the new compound **1** (Supplementary Information).

## Supplementary Information

Supplementary Information.
