# Supplementary material for: Flavonoids and alkaloids from the rhizomes of Zephyranthes ajax Hort. and their cytotoxicity
Source: Sci Rep. 2020 Dec 17;10:22193. doi: 10.1038/s41598-020-78785-2 (PMC7747562; doi:10.1038/s41598-020-78785-2)
Supplement: Supplementary file 1 — Supplementary Information. [file 41598_2020_78785_MOESM1_ESM.doc]

**Supplementary data**

**Flavonoids and alkaloids from the rhizomes of *Zephyranthes* *ajax* Hort. and their cytotoxicity**

Khan Viet Nguyen^1,3^, Duc Viet Ho^1^, Nhan Trong Le^1^, Kiem Van Phan^2^, Jyrki Heinämäki^3^, Ain Raal^3,*^, Hoai Thi Nguyen^1,*^

*^1^Faculty of Pharmacy, Hue University of Medicine and Pharmacy, Hue University, 06 Ngo Quyen, Hue City, Vietnam.*

*^2^Institute of Marine Biochemistry, The Vietnam Academy of Science and Technology, Hanoi, Vietnam.*

*^3^Institute of Pharmacy, Faculty of Medicine, University of Tartu, 1 Nooruse str., 50411 Tartu, Estonia*

**List of Supporting Information:**

**Figure S1.** ^1^H-NMR (500 MHz) spectrum of compound **1** in CD_3_OD.

**Figure S2.** ^1^H-NMR (500 MHz) spectrum of compound **1** in CD_3_OD (expansion 1).

**Figure S3.** ^1^H-NMR (500 MHz) spectrum of compound **1** in CD_3_OD (expansion 2).

**Figure S4.** ^13^C-NMR (125 MHz) spectrum of compound **1** in CD_3_OD.

**Figure S5.** DEPT spectrum of compound **1** in CD_3_OD.

**Figure S6.** HSQC spectrum of compound **1** in CD_3_OD.

**Figure S7.** HMBC spectrum of compound **1** in CD_3_OD.

**Figure S8.** HRESIMS spectrum of compound **1**.

**Figure S9.** CD spectrum of compound **1**.

**Figure S10.** UV spectrum of compound **1**.


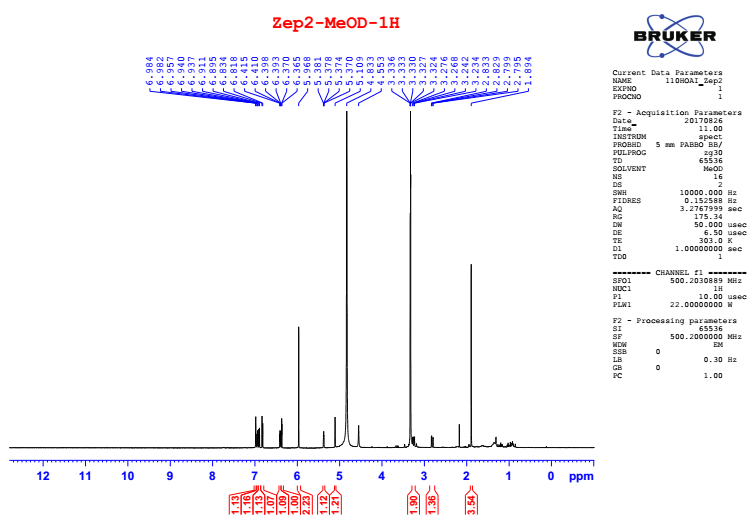


**Figure S1.** ^1^H-NMR (500 MHz) spectrum of compound **1** in CD_3_OD.


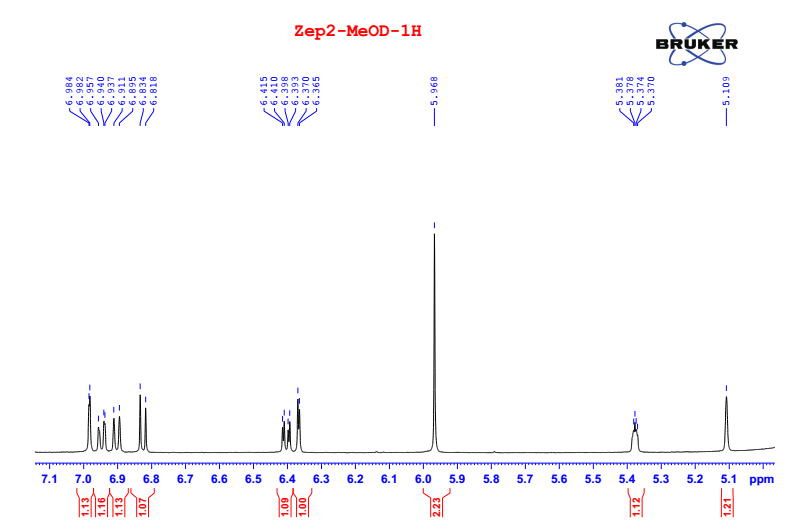


**Figure S2.** ^1^H-NMR (500 MHz) spectrum of compound **1** in CD_3_OD (expansion 1).


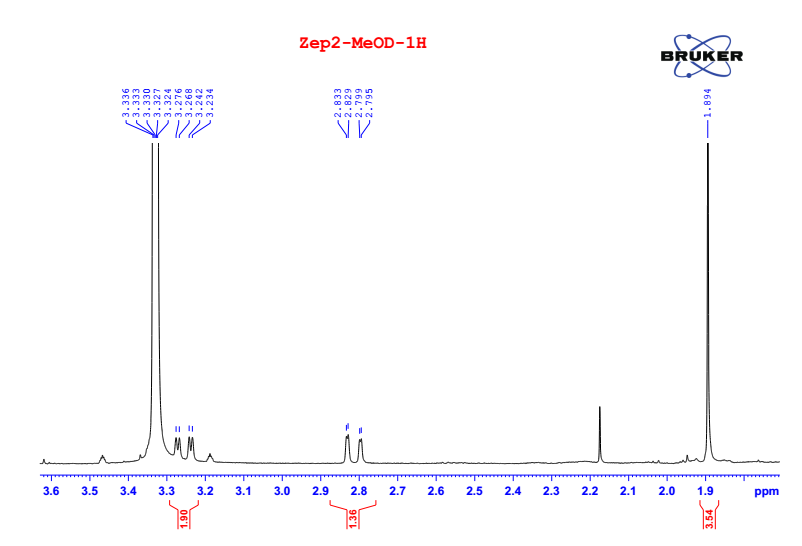


**Figure S3.** ^1^H-NMR (500 MHz) spectrum of compound **1** in CD_3_OD (expansion 2).


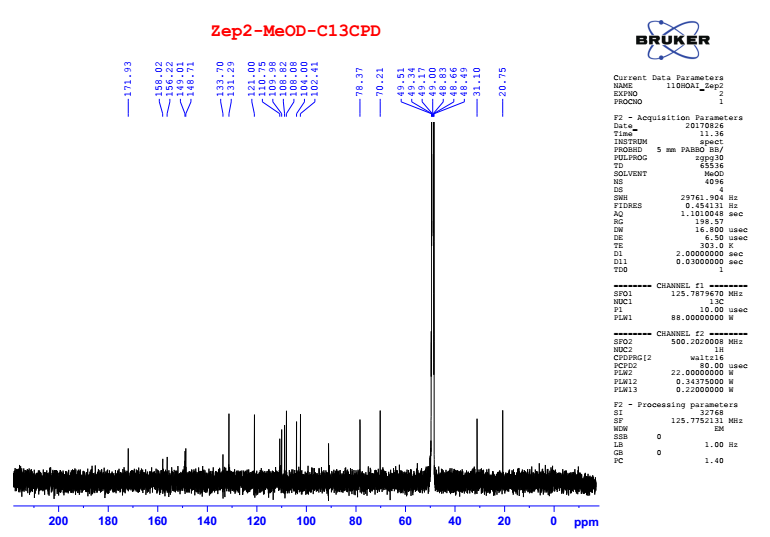


**Figure S4.** ^13^C-NMR (125 MHz) spectrum of compound **1** in CD_3_OD.


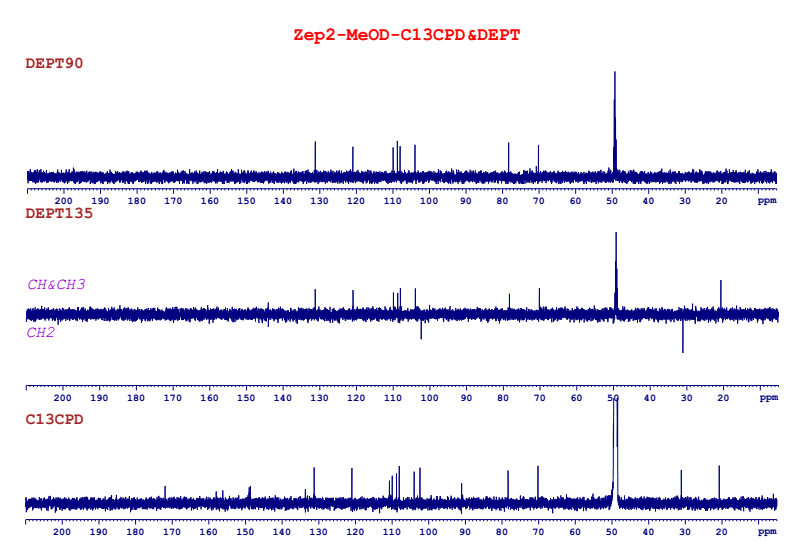


**Figure S5.** DEPT spectrum of compound **1** in CD_3_OD


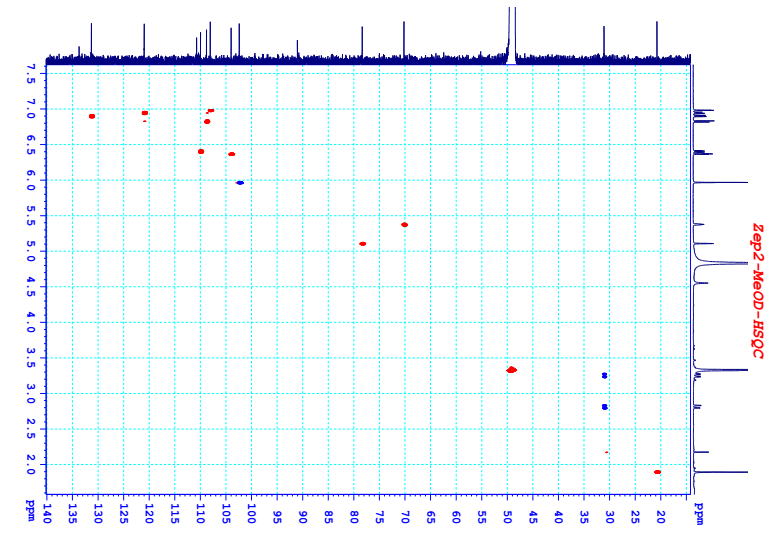


**Figure S6.** HSQC spectrum of compound **1** in CD_3_OD.


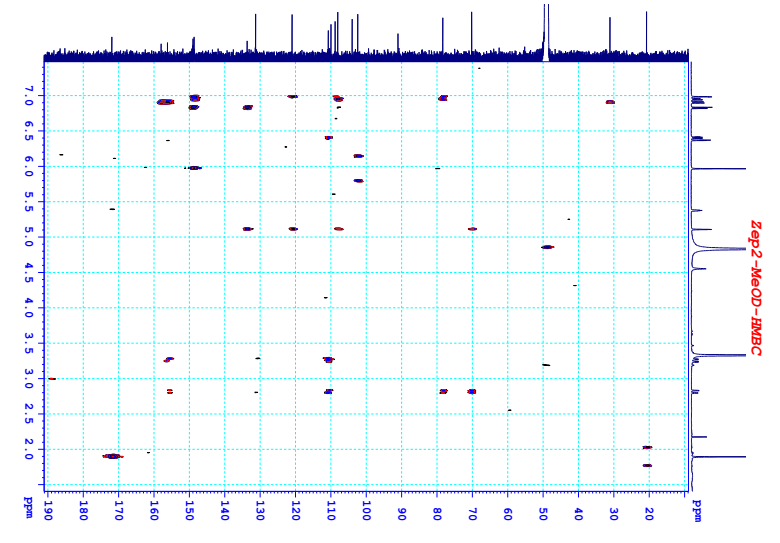


**Figure S7.** HMBC spectrum of compound **1** in CD_3_OD.

**Figure S8.** HRESIMS spectrum of compound **1**.


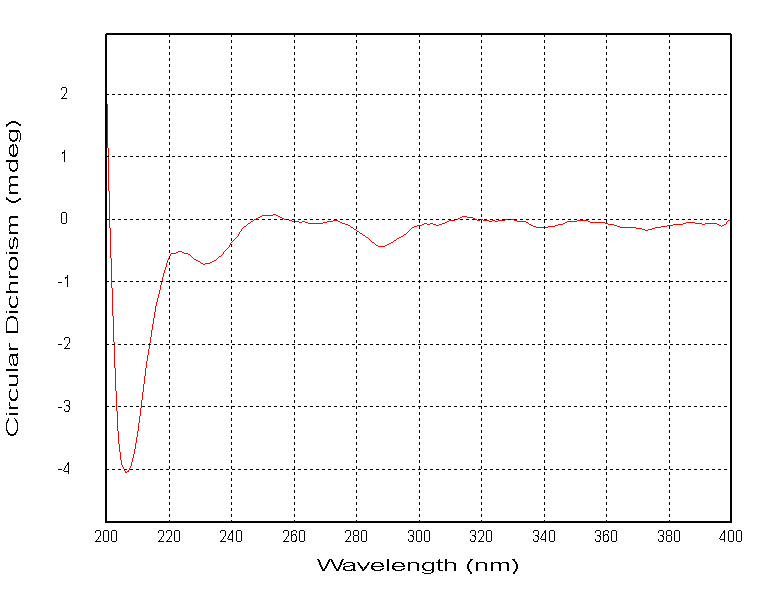


**Figure S9.** CD spectrum of compound **1**.


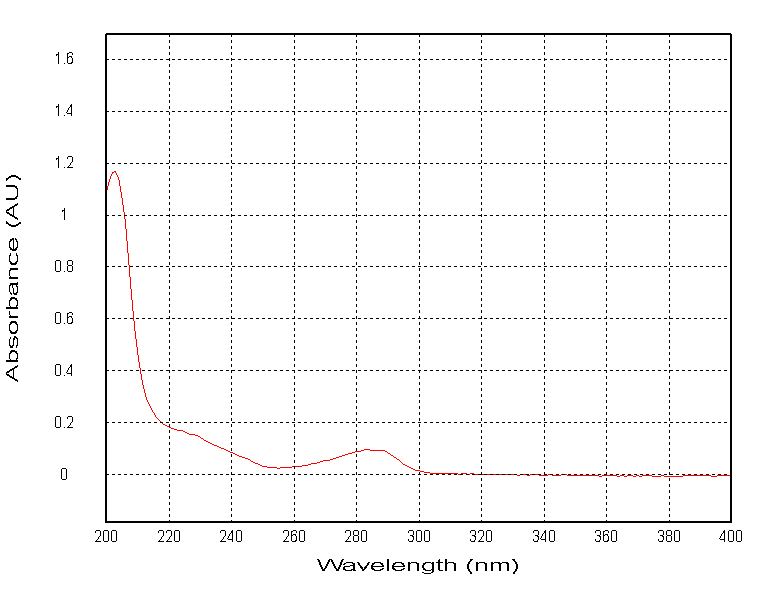


**Figure S10.** UV spectrum of compound **1**.
